# Supplementary material for: Analyzing human knockouts to validate GPR151 as a therapeutic target for reduction of body mass index
Source: PLoS Genet. 2022 Apr 5;18(4):e1010093. doi: 10.1371/journal.pgen.1010093 (PMC9022822; doi:10.1371/journal.pgen.1010093)
Supplement: S1 Table — (DOCX) [file pgen.1010093.s001.docx]

**S1 Table. List of GPR151 plof variants identified in 30,833 exomes and 9,292 genomes**

| **GRCh38 chr:pos** | **Reference**  **allele** | **Alternate**  **allele** | **Allele frequency** | **Heterozygous**  **carriers** | **Homozygous**  **carriers** | **Variant effect** | **HGVSc** | **HGVSp** |
| --- | --- | --- | --- | --- | --- | --- | --- | --- |
| 5:146516033 | G | T | 0.00003 | 2 | 0 | stop gained | c.81C>A | p.Tyr27Ter |
| 5:146515886 | CAG | C | 0.00017 | 14 | 0 | frameshift | c.226_227del | p.Leu76AspfsTer8 |
| 5:146515831 | G | A | 0.00111 | 85 | 2 | stop gained | c.283C>T | p.Arg95Ter |
| 5:146515817 | G | T | 0.01869 | 1394 | 40 | stop gained | c.297C>A | p.Tyr99Ter |
| 5:146515809 | CT | C | 0.00002 | 1 | 0 | frameshift | c.304del | p.Ser102ValfsTer5 |
| 5:146515741 | T | A | 0.00002 | 1 | 0 | stop gained | c.373A>T | p.Lys125Ter |
| 5:146515673 | CACTT | C | 0.00031 | 20 | 0 | frameshift | c.437_440del | p.Gln146ArgfsTer104 |
| 5:146515587 | CTA | C | 0.00245 | 184 | 6 | frameshift | c.525_526del | p.Phe175LeufsTer7 |
| 5:146515420 | G | A | 0.00008 | 5 | 0 | stop gained | c.694C>T | p.Arg232Ter |
| 5:146515145 | C | T | 0.00005 | 3 | 0 | stop gained | c.969G>A | p.Trp323Ter |
| 5:146515116 | G | C | 0.00005 | 3 | 0 | stop gained | c.998C>G | p.Ser333Ter |

*chr, chromosome; pos, position; HGVSc and HGVSp, Human Genome Variation Society coding or protein level change, respectively*
