# Supplementary material for: Analyzing human knockouts to validate GPR151 as a therapeutic target for reduction of body mass index
Source: PLoS Genet. 2022 Apr 5;18(4):e1010093. doi: 10.1371/journal.pgen.1010093 (PMC9022822; doi:10.1371/journal.pgen.1010093)
Supplement: S2 Table — (DOCX) [file pgen.1010093.s002.docx]

**S2 Table. *GPR151* association with waist-to-hip ratio and lipid-related biomarkers**

| **GRCh38 chr:pos** | **Reference**  **allele** | **Alternate**  **allele** | **Phenotype** | **HGVSp** | **Genotype counts (RR\|RA\|AA)** | **P-value** | **Beta [95% CI] (additive)** |
| --- | --- | --- | --- | --- | --- | --- | --- |
| plof Gene Burden |  |  | Cholesterol  (mg / dl) |  | 35888\|1599\|45 | 0.242 | 1.414  [-0.957 - 3.786] |
| 5:146515831 | G | A |  | Arg95Ter | 37452\|78\|2 | 0.740 | 1.790  [-8.777 - 12.357] |
| 5:146515817 | G | T |  | Tyr99Ter | 36178\|1316\|38 | 0.598 | 0.700  [-1.9 – 3.3] |
| 5:146515587 | CTA | C |  | Phe175LeufsTer7 | 37356\|171\|5 | 0.383 | 3.161  [-3.94 – 10.26] |
| plof Gene Burden |  |  | Triglycerides  (mg / dl) |  | 35742\|1591\|45 | 0.925 | 0.274  [-5.384 – 5.93] |
| 5:146515831 | G | A |  | Arg95Ter | 37297\|79\|2 | 0.581 | 7.060  [-18.014 – 32.133] |
| 5:146515817 | G | T |  | Tyr99Ter | 36033\|1307\|38 | 0.855 | -0.580  [-6.784 – 5.623] |
| 5:146515587 | CTA | C |  | Phe175LeufsTer7 | 37202\|171\|5 | 0.862 | -1.507  [-18.44 – 15.42] |
| plof Gene Burden |  |  | Waist-to-Hip Ratio |  | 28540\|1263\|42 | 0.567 | -0.001  [-0.004 – 0002] |
| 5:146515831 | G | A |  | Arg95Ter | 29787\|56\|2 | 0.147 | 0.010  [-0.004 – 0.024] |
| 5:146515817 | G | T |  | Tyr99Ter | 28773\|1035\|37 | 0.222 | -0.002  [-0.005 – 0.001] |
| 5:146515587 | CTA | C |  | Phe175LeufsTer7 | 29698\|144\|3 | 0.844 | -0.001  [-0.010 – 0.008] |

*chr, chromosome; pos, position; HGVSp, Human Genome Variation Society protein level change; R, reference allele; A, alternate allele; kg, kilograms; m, meter; CI, confidence interval; mg/dl, milligrams per deciliter*
