# Supplementary material for: Analyzing human knockouts to validate GPR151 as a therapeutic target for reduction of body mass index
Source: PLoS Genet. 2022 Apr 5;18(4):e1010093. doi: 10.1371/journal.pgen.1010093 (PMC9022822; doi:10.1371/journal.pgen.1010093)
Supplement: S3 Table — (DOCX) [file pgen.1010093.s003.docx]

**S3 Table. *GPR151* association with MI**

| **GRCh38 chr:pos** | **Reference**  **allele** | **Alternate**  **allele** | **HGVSp** | **Genotypes cases**  **(RR\|RA\|AA)** | **Genotypes controls**  **(RR\|RA\|AA)** | **P-value** | **OR [95% CI]** | **OR [95% CI]**  **(knockouts only)** |
| --- | --- | --- | --- | --- | --- | --- | --- | --- |
| 5:146515831 | G | A | Arg95Ter | 20690\|50\|2 | 19343\|35\|0 | 0.33 | 1.39  [0.71 – 2.72] |  |
| 5:146515817 | G | T | Tyr99Ter | 19992\|736\|14 | 18694\|658\|26 | 0.90 | 0.99  [0.89 – 1.10] | 0.81  [0.38 – 1.71] |
| 5:146515587 | CTA | C | Phe175LeufsTer7 | 20638\|100\|4 | 19292\|84\|2 | 0.44 | 0.90  [0.68– 1.18] |  |
| Gene Burden |  |  |  | 19809\|913\|20 | 18561\|789\|28 | 0.5 | 1.04  [0.39– 2.76] | 1.11  [0.5 – 2.4] |

*chr, chromosome; pos, position; HGVSp, Human Genome Variation Society protein level change; R, reference allele; A, alternate allele; OR, odds ratio, CI, confidence interval*
