# Supplementary material for: Analyzing human knockouts to validate GPR151 as a therapeutic target for reduction of body mass index
Source: PLoS Genet. 2022 Apr 5;18(4):e1010093. doi: 10.1371/journal.pgen.1010093 (PMC9022822; doi:10.1371/journal.pgen.1010093)
Supplement: Attachment — Submitted filename: Gurtan et al, response to reviewers.pdf [file pgen.1010093.s005.pdf]

<u>Response to reviewers</u>

We thank the reviewers for their helpful comments and questions. We were pleased to read that the reviewers found our study to be "a careful and thorough paper" with "functional analyses" that "are vitally important" and may "save millions of dollars in wasted preclinical drug discovery efforts" (Reviewer #1); "clear, concise" and "interesting" (Reviewer #2); and "well written" with results that "are of great interest for the community" as "it is crucial to publish negative results" (Reviewer #3).

We have revised our manuscript substantially to address all reviewers' points. We have:

1. Included additional analysis of PGR data to strengthen our conclusions.
2. Meta-analyzed the UK Biobank data to confirm absence of a body mass index (BMI) effect in *GPR151* knockouts.
3. Meta-analyzed GIANT Consortium data to replicate the original published findings.
4. Revised the text, tables and figures throughout for content and clarity.

Below, we list our revisions point-by-point.

Reviewer #1, Eric Vallabh Minikel:

1.1 This is a careful and thorough paper on an important topic. Functional analyses of GWAS hits like these are vitally important and the results reported here will probably save millions of dollars in wasted preclinical drug discovery efforts. The science is well done and paper well-written, I have no major issues with anything here.

- **We thank the reviewer for his comments. We believe that our study's strength lies in its synthesis of experimental functional data with genetic observations.**

1.2 In the introductory paragraph beginning "G protein coupled receptors (GPCRs) are attractive drug targets…" it might be nice to give some background for the reader on anything known about GPR151 in particular. I looked it up in GTEx and was surprised to see it expressed at appreciable levels only in brain. Perhaps none of this matters since you found it's not associated with BMI anyway, but for me as an outsider who has never thought about this gene or this phenotype before, any background you could give would be helpful.

- **We agree that the paper would benefit from a description of GPR151, including its expression. We have added the following text to the revision:**
  - **Introduction: "GPR151 is a poorly understood, brain-specific GPCR (Sjostedt et al. 2020) for which loss-of-function has been associated with decreased BMI (Emdin et al. 2018; Tanigawa et al. 2019; Akbari et al. 2021)."**
  - **Results and Discussion: "In human, pig, and mouse, GPR151 is expressed primarily in the brain (proteinatlas.org; Sjostedt et al. 2020). In mouse, *Gpr151* is expressed in the habenula, which is located in the dorsal thalamus of the brain (Broms et al. 2015). In humans, *GPR151* is expressed in the midbrain (proteinatlas.org; Sjostedt et al. 2020)."**

1.3 I kept looking for evidence that the protein is not expressed in the KO mice. Then I realized that the use of HA tag in the in vitro experiments and the use of a riboprobe in the mouse sections must be because there simply exists no good antibody for this protein. If so, this fact might be stated up front to avoid the reader wondering. A quick google search indicates some vendors claim to sell GPR151 antibodies; if the authors already tested these and found them not to work, that info might be useful to some readers.

- **To address this point, we have added the following text to the revised manuscript:**
  - **Results and Discussion: "Changes in GPR151 protein sequence may alter epitopes detected by antibodies specific to GPR151 and thus confound detection by**

western blot. Therefore, constructs were tagged at the N-terminus with an HA epitope tag to directly compare expression of GPR151 protein variants *in vitro*."

1.4 I found myself wondering how the originally reported association could so spectacularly fail to replicate. Two possibilities came to mind. One, the UKBB association was right at the edge of significance (4.9e-8 in Emdin 2018, 5.7e-9 in Akbari 2021) — was it just a false positive? Two, is there any chance that there is some real effect on BMI but only through an interaction with age or some other variable? How does the median age in this cohort compare to that in UKBB? Might the KO mice have ended up with lower BMI at some age greater than 17 weeks? I agree with the authors that the data already presented here are pretty much sufficient to kill any interest in this gene as drug target, but some discussion/caveats on this topic might be nice to have.
- **These questions, also raised by Reviewer #2, are answered in points 2.5 and 2.6 below.**

Reviewer #2, Anonymous:

2.1 Overall the manuscript is clear, concise and makes an interesting point.
- **We thank the reviewer for these comments.**

2.2 Abstract – The authors state "Moreover, loss of GPR151 confers a nominally significant increase in risk of T2D (odds ratio = 1.2, p value = 0.03). Relative to wild-type mice, Gpr151-/- animals exhibit no difference in body weight on normal chow, and higher body weight on a high-fat diet, consistent with the findings in humans." Firstly, the authors state the association with increased risk of T2D is nominally significant but this does not take into account the number of tests done (BMI, T2D, cholesterol, triglycerides, waist-hip ratio, MI) so I think this result as stated risks over-interpreting the data (additional comments on T2D and other analysis in other points below).
- **As suggested by the reviewer, we took into account the number of tests, after which the association with T2D did not meet the threshold of significance. We have revised the text:**
    - **Results and Discussion: "For the plof gene burden we observed a slight increase in T2D (P=0.03, OR = 1.18 [1.02 – 1.37]), but this increase did not meet the threshold of significance corrected for multiple testing (threshold of p = 0.0083)."**

2.3 Secondly, the authors state that knockout mice have higher body weight on a high-fat diet, and that this is consistent with human findings. But this is confusing as the initial human data suggested that lof variants in GPR151 lowered BMI in humans and the authors here with their own data show that they do not see any evidence for differences in BMI in humans with lof, so unclear what is meant by being consistent with findings in humans.
- **We originally meant to point out that the lack of reduction in body weight was consistent with humans but realize now that our original statement was unclear. We have revised the abstract and text for clarity:**
    - **Abstract: "We test if *GPR151* plof is associated with BMI, T2D, or other metabolic traits and find that *GPR151* deficiency in complete human knockouts is not associated with clinically significant differences in these traits. Relative to *Gpr151*$^{+/+}$ mice, *Gpr151*$^{-/-}$ animals exhibit no difference to body weight on normal chow and higher body weight on a high-fat diet."**
    - **Results and Discussion: "The body weights of male and female *Gpr151*$^{-/-}$ mice were indistinguishable from *Gpr151*$^{+/+}$ control mice on a standard chow diet and were elevated in male *Gpr151*$^{-/-}$ mice on a high fat diet without a corresponding increase in food intake. The preclinical model data indicate that the lack of association with BMI is generalizable rather than a human population-specific phenomenon."**

2.4 Background/ context of the findings - Some of the introduction is missing out key recent papers such as Sobreira et al., 2021 relating to the effects at the FTO locus in nearby loci. Also the statement

regarding melanocortin 4 receptor agonists is somewhat misleading. Though many of these compounds do have undesirable cardiovascular effects this is not true of all, and so the phrase should be re-stated. Setmelanotide seems to be well tolerated with minimal side effects, this is published in e.g. Clement et al 2018; Haw et al., 2020 and indeed the review cited by the authors Yeo et al 2021 shows this clearly in table 3. I think it is still valid to state additional body weight reduction drugs are needed but important to ensure the statements made are correct.

- **We have revised the introduction to incorporate the reviewer's suggestions. We now cite Sobreira et al. We have refined our description of MC4R agonists:**
  - **Introduction: "MC4R modulators were often associated with adverse cardiovascular side effects until the identification of setmelanotide, which does not elicit these undesirable effects and was approved in the United States and Europe for treatment of genetic obesity (Yeo et al. 2021)."**

2.5 An important point which the authors do not make is that even in the original publications describing an association between pLOF variant Arg95Ter, the effect on BMI was very modest (−0.36 kg/m2). So arguably, the expectation would be that this effect would be too modest to have meaningful clinical impact, although I acknowledge the previous papers were mostly focused on additive effects. However, there are 20 homozygous carriers for this variant in biobank (and only one is the authors data) and the authors could easily investigate this and combine it in meta-analysis with their own data, which I do think is warranted to gain clarity as to the effect of this variant on BMI.

- **We agree with the reviewer that (i) the main focus of our manuscript is analysis of human knockouts, and (ii) prior publications used additive genetic analyses and were not adequately powered to conduct analyses in human knockouts. As suggested by the reviewer, we conducted a meta-analysis and found no significant association.**
- **We have included this analysis in our revision:**
  - **Results and Discussion: "We analyzed knockouts across all variants versus reference carriers and did not observe significant association for either the gene burden result (knockout n = 38, p = 0.98) or Tyr99X variant (n = 34, p = 0.55). We also performed a sample size-based meta-analysis with UK Biobank GPR151 knockouts (n = 28) reported previously (Tanigawa et al. 2019). The meta-analyzed p-value remained non-significant (p = 0.67)."**

2.6 (a) Is there evidence for replication/lack of replication of an association between the previous pLOF variant (Arg95Ter) at this locus and reduced BMI? I note that here the authors show that their results are still consistent (CIs overlap) with a protective effect of this variant on BMI (their numbers here are smaller than previously published so lack power). I would suggest a meta-analysis of this variant across all available cohorts with this data is warranted as the data shown here do not provide evidence "against" this association. The authors might consider including data from non overlapping previous datasets and if possible the Genes and Health initiative as another effort enriched for autozygous individuals. (b) Is there evidence that complete loss of function at this locus will have a clinically meaningful effect on BMI in humans? The two questions are not exactly identical because although the authors do not have evidence for a clinically meaningful reduction in BMI in their population, their data do not refute an association between the Arg95Ter and reduced BMI. Indeed their CIs overlap previous effect estimates with larger sample sizes. So I think a meta-analysis across all datasets with this variant is warranted to try and establish whether the original association stands, or not. Regarding the second point one might argue even if the effects replicate the effect on BMI overall is modest. Again I think given the available data in UK biobank including additional homozygous pLOF carriers this point would be best addressed by meta-analysing the results across all possible datasets the authors can access, they clearly have access to UK biobank so this should be straightforward. I'd suggest if possible including data from Genes and Health would also be interesting and add value. Importantly if the desire is to include only data from null alleles it would be critical to ensure incomplete loss of function variants are not included in the burden test.

- **The main goal of our paper was to assess if there is a clinically meaningful effect in human knockouts. We did not observe a clinically meaningful effect size in CNCD, despite a sizeable number of knockouts in PGR. The original published observations indicated a weak effect size using an additive model. Based on the reviewer's suggestions, we analyzed the GIANT consortium and replicated the original finding that Arg95Ter is associated with a weak BMI effect. We could not analyze the Genes and Health study because its effect estimates are not publicly available. Meta-analysis of *GPR151* knockouts in UK Biobank is described in point 2.5 above. The new analyses are included in our revised manuscript:**
    - **Results and Discussion: "As stated above, in PGR alone or in the combined meta-analyses, we did not observe a clinically meaningful effect on BMI in human knockouts despite a sizeable number of plof homozygous carriers for *GPR151*. We further examined if the previously reported weak genetic effect on BMI, largely conferred by heterozygous plof carriers, is reproducible. We meta-analyzed our results with summary statistics from the GIANT consortium (Turcot et al. 2018) (Total N = 497,110; African Ancestry = 27,610; Admixed American Ancestry = 10,772; East Asian Ancestry = 8,839; European Ancestry = 449,889). With a significantly larger sample size, we replicated the Arg95Ter association with BMI (p = 6.72E-4; Beta = -0.042 [-0.063 - -0.0171]) with an additive model as used in the published study. There was no evidence of a population or study-specific effect (p-value for heterogeneity = 0.95). Hence, the original additive association between Arg95Ter and BMI, based primarily on heterozygous plof carriers, is reproducible but with a weak effect. Our findings with human knockouts across multiple plof variants indicate that complete absence of GPR151 does not further enhance this weak effect into a therapeutically meaningful reduction in BMI."**

2.7 Looking at the data in Table 1, the second termination variant also has an effect size point estimate that is consistent with lower BMI for homozygous carriers. So I think there is a real question whether the frameshift variant which occurs much later in the protein is fundamentally different. Indeed the author's data show that this variant is expressed although at low levels. The authors conclude this variant is loss of function because of its lower levels of expression but two bands are clearly seen so the variant is expressed, and there is no in vitro functional data to support the statement that this is a complete loss of function variant. Given this, it would be good to see the gene burden test results removing this variant from the burden test.

- **To address the severity of the frameshift variant, we have added the following text:**
    - **Results and Discussion: "Phe175LfsTer7 is missing the last three of the protein's seven transmembrane domains, the entire cytoplasmic tail, and intracellular loop 3, which is typically critical for G protein activity. The severity and diminished expression of this truncation indicate that this variant is a loss-of-function. Our *in vitro* observations confirm that the homozygous *GPR151* plof variants in PGR are loss-of-function alleles."**

2.8 The association with T2D is nominal only and not adjusted for the different tests and phenotypes looked at, so I think interpretation needs to take this into account. Specifically, and if the authors remove the frameshift variant it looks like their CIs overlap the previous estimates for a protective effect? Instead of a straight power calculation for what the authors are powered to detect, I would prefer to see a power calculation for what effect sizes the authors are powered to rule out? Again I think combining this new data with previously published data in meta-analysis would increase power and provide more clarity as to what the data are showing.

- **We provide power calculations to determine the effect size we can detect. Additionally, the nominal association lost significance after accounting for multiple testing. We have revised the text:**

- **Results and Discussion: "For gene-burden analyses, our study was powered to observe an odds ratio of T2D of 0.82 or lower (80% at an α = 0.05). No individual plof variant was associated with a significant change in the risk for T2D. For the plof gene burden we observed a slight increase in T2D risk (P=0.03, OR = 1.18 [1.02 – 1.37]), but this increase did not meet the threshold of significance corrected for multiple testing (threshold of p = 0.0083)."**

2.9 The mouse data suggest a possible sexual dimorphism in the phenotype of the knockout mice, have the authors analysed the human data stratified by sex? I think despite smaller numbers and loss of power it would be interesting to check whether there is any evidence from human data for different variant effects between the sexes.

- **We meta-analyzed plofs observed in PGR with the Arg95Ter variant in UK Biobank separately for males (-0.28 [-0.57 to -0.21] kg/m^2) and females (-0.39 [-0.43 - -0.12] kg/m^2). The confidence intervals were overlapping and there was no evidence of a dimorphic effect (pvalue heterogeneity = 0.33).**

2.10 The manuscript is missing a discussion on how the authors interpret their results in light of previous association results with reduced BMI and obesity at this gene in much larger sample sizes (including comparable numbers for some homozygous individuals and variants)? Specifically, in three different cohorts a burden of pLOF and missense predicted deleterious variants associated with reduced BMI. How do the authors interpret their data in light of previous findings? I think a meta-analysis across available datasets may help provide further clarity here.

- **Based on meta-analyses we added to the manuscript to address multiple related comments from reviewers, we draw the following conclusion added to our revised text:**
  - **Results and Discussion: "As stated above, in PGR alone or in the combined meta-analyses, we did not observe a clinically meaningful effect on BMI in human knockouts despite a sizeable number of plof homozygous carriers for *GPR151*. We further examined if the previously reported weak genetic effect on BMI, largely conferred by heterozygous plof carriers, is reproducible. We meta-analyzed our results with summary statistics from the GIANT consortium (Turcot et al. 2018) (Total N = 497,110; African Ancestry = 27,610; Admixed American Ancestry = 10,772; East Asian Ancestry = 8,839; European Ancestry = 449,889). With a significantly larger sample size, we replicated the Arg95Ter association with BMI (p = 6.72E-4; Beta = -0.042 [-0.063 - -0.0171]) with an additive model as used in the published study. There was no evidence of a population or study-specific effect (p-value for heterogeneity = 0.95). Hence, the original additive association between Arg95Ter and BMI, based primarily on heterozygous plof carriers, is reproducible but with a weak effect. Our findings with human knockouts across multiple plof variants indicate that complete absence of GPR151 does not further enhance this weak effect into a therapeutically meaningful reduction in BMI."**

2.11 Data availability: I could not find a specific data availability statement in the manuscript aside from a "no-some restrictions will apply" in the box at the front. Please clarify exactly what data will be available and how, for example is the mouse line available from somewhere, will the summary statistics for all GPR151 variants and associated phenotypes analyzed in the manuscript be available somewhere? Although the authors mention all associated data is within the manuscript this is not really the case as full genotype counts for ref/ref ref/alt and alt/alt and corresponding phenotypes are not given for every phenotype tested. If some data have restricted access please explain what data cannot be made available and why.

- **The missing data (for example, reference and alternate allele counts) have been added to the manuscript. All relevant mouse data are included in the manuscript. The *Gpr151***

knockout mice are available with a Materials Transfer Agreement (MTA). Access to individual-level patient phenotype and genotype data is restricted.

2.12 In all the tables, for clarity it would be helpful to see the N total in cases / controls or in the entire test data not just hets and hom carriers, or better still the number of each genotype class in cases and controls separately.

- **We have added this information to the relevant tables.**

2.13 There is no author summary provided.

- **We have added an author summary:**
    - **"Human genetics studies can provide compelling targets for therapeutic intervention. While some therapeutic targets, such as *PCSK9*, are based on extensive genetic validation, many others are based on weaker associations with variants of unknown consequence that require further validation. Recent publications reported associations between loss of *GPR151* function and low body mass index (BMI), raising the possibility of inhibiting *GPR151* for the treatment of obesity and metabolic syndromes. To evaluate the relationship between *GPR151* and BMI, we (1) identified and experimentally confirmed loss-of-function variants present in the Pakistan Genome Resource (PGR) biobank, one of the world's largest biobanks of human gene "knockouts", (2) analyzed these loss-of-function variants individually and in burden tests for association with BMI and other metabolic traits or diseases, and (3) verified the evolutionary conservation of our findings in mice lacking *Gpr151*. We observe that *GPR151* loss does not affect BMI to a clinically relevant extent and conclude that inhibiting *GPR151* may not be effective at treating obesity."**

2.14 Figure 2 - I suggest modifying Figure 2 title to better represent entire multipanel figure or pulling out the weight curves into separate figure. Would encourage authors to change the colour from male and female mice away from stereotypes of blue and pink.

- **We have revised the manuscript as suggested by the reviewer:**
    - **We split Figure 2 into two figures:**
        - **Figure 2: *Gpr151*$^{-/-}$ mice do not express *Gpr151* mRNA.**
        - **Figure 3: Male *Gpr151*$^{-/-}$ mice gain weight on high fat diet.**
            - **In this figure, we have changed the colors for males and females to green and orange, respectively.**

2.15 Figure 2D the riboprobe for GPR151 intestine data in wild-type is not particularly obvious and its detection seems a little subjective.

- **We have added a magnified inset in Figure 2 to facilitate seeing the riboprobe signal.**

2.16 Methods: "We obtained a list of high-quality protein coding transcripts with annotated start and stop codons." Please clarify where this was obtained from or how exactly is a high-quality protein coding transcript defined?

- **We have clarified this point in the revised text:**
    - **"For human *GPR151*, we used Ensembl Transcript ENST00000311104, which is the only annotated transcript for this gene."**

2.17 Methods: Case classification, T2D cases “1) Documented history of diabetes” , please specify what documented history of diabetes means? Also, how was type 1 diabetes, excluded?

- **We have added this information to the revised text:**
    - **Materials and Methods, Case classification: “Patients were categorized as T2D cases if they satisfied any one of the following criteria: (1) Physician diagnosis at a diabetes clinic, (2) HbA1c > 6.5%, (3) use of glucose lowering medication or (4) fasting glucose > 126 mg/dl. An age of first diagnosis >22 years was used to exclude type 1 diabetes.”**

2.18 Methods: adjusted for top 5 principal components, why 5?

- **The first 5 PCs account for batch effects as well as local population structure. In our prior publications, we had conducted analyses using a scree plot to identify the number of PCs most information in our study and we found that the first 5 PCs explained most of the variability. Keeping 10 makes no difference in the overall results.**

2.19 Unclear whether genomes and exomes were treated the same or whether there was any adjustment for batch effect in the analysis? It looks like the data were analysed separately and then meta-analysed but it would be good to make this a bit clearer.

- **We have clarified this point in the revised text:**
    - **Materials and Methods: “The genomes and exomes datasets were analyzed separately and the summary statistics were meta-analyzed using inverse variance weighted meta-analysis as implemented in METAL (Willer et al. 2010).”**

2.20 Methods : somewhat unclear the authors state that “At six-weeks of age, Gpr151-/- mice and wild type littermates were individually housed for body weight and food consumption measurements and provided either a standard chow diet (Purina Picolab 5053) or high fat diet in which 60% kcal is derived from fat (Research Diets D12492i) with ad libitum access to water.” But in the following sentence it is stated that female mice were group housed, so were they group housed from the outset and then from six-weeks the male Kos and wild-type only were individually housed?

- **We have clarified this point in the revised manuscript:**
    - **Materials and Methods, Generation and phenotyping of *Gpr151*$^{-/-}$ mice: “At five weeks of age, male *Gpr151*$^{+/+}$ and *Gpr151*$^{-/-}$ littermates were split from group housing to individual housing to monitor body weight and food consumption. Female mice remained group housed for body weight studies.”**

Reviewer #3, Amelie Bonnefond:

3.1 This article is well written and the reviewer believes that these negative results are of great interest for the community (it is crucial to publish negative results).

- **We thank the reviewer for the comments. We agree that there is value to publishing the absence of a therapeutically meaningful association.**

3.2 If the reviewer is right, the authors actually combined carriers of heterozygous and homozygous LOF GPR151 variants for their burden analysis, while the abstract and introduction mainly tackled “human homozygous loss-of-function”. It would be important to also analyze carriers of homozygous LOF GPR151 variants only (and remove the carriers of heterozygous variants). Furthermore, to enhance the statistical power of this analysis, the authors could combine the carriers of the three LOF variants (at homozygous state).

- **This analysis was conducted and is described in the text:**
    - **Results and Discussion: “Most importantly, homozygous *GPR151* knockout did not confer low BMI compared to non-carriers. We analyzed knockouts across all variants versus reference carriers and did not observe significant association for**

either the gene burden result (knockout n = 38, p = 0.98) or Tyr99X variant (n = 34, p = 0.55)"

3.3 The mean depth of coverage of GPR151 in the Pakistan Genome Resource should be provided, as well as the genotyping success rate for the three variants.

- **We have included this information in the revised text:**
  - **Materials and Methods, Variant quality control (QC) and annotation: "Samples were sequenced at an average of 30X coverage."**
  - **Materials and Methods, Variant quality control (QC) and annotation: "The three reported homozygous *GPR151* variants (Arg95Ter, Tyr99Ter, and Phe175LeufsTer7) have a call rate of 1 (i.e. zero missingness)."**

3.4 The authors should also analyse the effect of LOF variants (in homozygous carriers) on obesity risk (obese participants versus normal weight participants)

- **We conducted this analysis and observed no association with obesity status.**

	**Obese (> 30 BMI)**	**Not Obese**
**WT**	**2006**	**16092**
**KO**	**3**	**23**

- **Fisher p-value = 0.95**
